# aMMP-8 and MMP-9: Potential Biomarkers for periodontitis Progression in Patients Receiving Radiotherapy for Head and Neck Carcinoma

**DOI:** 10.1177/10732748251351419

**Published:** 2025-06-22

**Authors:** Ella Brandt, Wendy Kaman, Floris Bikker, Ismo T. Räisänen, Antti Mäkitie, Mutlu Keskin, Didem Karaçetin, Jaana Hagström, Jaana Rautava, Timo Sorsa

**Affiliations:** 1 Department of Oral and Maxillofacial Diseases, University of Helsinki and Helsinki University Hospital, Finland; 2 Department of Oral Biochemistry, Academic Centre for Dentistry Amsterdam, University of Amsterdam and VU University Amsterdam, Netherlands; 3 Department of Otorhinolaryngology—Head and Neck Surgery, University of Helsinki and Helsinki University Hospital, Finland; 4 Research Program in Systems Oncology, Faculty of Medicine, 3835 University of Helsinki, Finland; 5 Department of Periodontology, Faculty of Dentistry, Istanbul University-Cerrahpaşa, Istanbul, Turkey; 6 Department of Radiation Oncology, 420479 Biruni University, Istanbul, Turkey; 7 Neolife Medical Center, Istanbul, Turkey; 8 HUS Diagnostics, Department of Pathology, University of Helsinki and Helsinki University Hospital, Finland; 9 Department of Oral Pathology and Radiology, University of Turku, Finland; 10 Department of Medicine and Dental Medicine, Karolinska Institute, Stockholm, Sweden

**Keywords:** matrix metalloproteinase, head and neck carcinoma, radiotherapy, periodontitis, biomarker

## Abstract

**Introduction:**

Matrix metalloproteinases (MMP)-8 and −9 are regarded as biomarkers for periodontitis. The impact of head and neck carcinoma (HNC) radiotherapy (RT) on these biomarkers remains largely unexplored. This study aims to evaluate the impact of HNC RT on the proteases from host and bacteria, and their associations with the progression of periodontitis.

**Methods:**

Twenty-one patients with HNC receiving RT were included in this study. Mouthrinse samples were collected before RT (T0) and at the end of RT (T1). Periodontal examinations were conducted at T0 and one month after RT (T2). Samples were analyzed using a point-of-care kit for active (a) MMP-8, and ELISA for total (t) MMP-8, MMP-9, and interleukin (IL)-6 levels. Molecular forms of MMP-9 were assessed by gelatinolytic zymography. The activity levels of bacterial proteases, gingipain from *Porphyromonas gingivalis* and dentilisin from *Treponema denticola*, were assayed by using Fluorescence Resonance Energy Transfer (FRET) peptide substrates. Modified cumulative risk score (CRS) index was used for combinatory biomarker analysis. We evaluated the impact of RT on biomarker concentrations as well as their associations with the mean clinical attachment loss (CAL) development, categorized as either progressive (CAL ≥0.1 mm) or low/no progression (CAL <0.1 mm).

**Results:**

All patients suffered from periodontitis. There were significant increases in the levels of aMMP-8, tMMP-8, aMMP-9, as well as in four modified CRS-indices due to RT (*P* < 0.05), but no impact on the activities of gingipain (*P* = 0.365) or dentilisin (*P* = 0.620). The mean levels of aMMP-8 and tMMP-9 were associated with CAL progression (*P* = 0.044, *P* = 0.029, respectively).

**Conclusion:**

HNC RT may result in the progression of periodontitis by increased activities of MMP-8 and -9. It seems that HNC RT has little impact on the activities of the bacteria-derived proteases dentilisin and gingipain.

## Plain Language Summary

This study evaluated the effect of head and neck cancer (HNC) radiotherapy (RT) on certain biomarkers that indicate gum disease, known as periodontitis. Among these biomarkers, MMP-8 and MMP-9 are proteins that cause tissue breakdown in periodontitis. We collected mouthrinse samples from 21 patients with HNC before and after RT to measure levels of these biomarkers, along with proteins from bacteria. The study found that RT increased the levels of MMP-8 and MMP-9 and were linked to the clinical worsening of periodontitis. However, the activities of bacterial proteins, dentilisin and gingipain, weren’t affected by RT. These results suggest that RT speeds up periodontitis, possibly through increased MMP levels. Therefore, a simple aMMP-8 test could help to predict periodontitis progression in patients with HNC receiving RT.

## Introduction

Head and neck carcinomas (HNCs) are common neoplasms with an annual incidence of nearly 900,000 new cases.^1^ Radiotherapy (RT) plays a crucial role in their treatment, either used alone or in combination with surgery and/or chemotherapy. Despite advances in the technique, RT has multiple side effects to oral health, such as acute damage in oral mucous membranes and decreased salivary flow.^2^ These side effects cause discomfort for the patient and create ideal conditions for bacterial growth and colonization in the oral cavity.^3^ From a dental perspective, the most significant late side effect of RT in the head and neck region is dental caries. However, periodontal complications, such as clinical attachment loss (CAL), may occur, as well.^4,5^

Periodontitis is an inflammatory condition characterized by breakdown of tooth-supporting tissues in response to infection caused by oral bacteria. Both host- and bacteria-derived proteolytic enzymes, known as proteases, along with cytokines, play significant roles in the pathogenesis of periodontitis.^6-8^ Matrix metalloproteinases (MMPs) are key enzymes that degrade extracellular matrix proteins and regulate immune responses.^9^ Among them, MMP-8 and -9 are the most abundant in the periodontium and are primarily responsible for periodontal tissue deterioration.^10^ Elevated levels of MMP-8 and MMP-9 have been identified in the oral fluids of individuals with periodontitis, indicating their potential as key biomarkers for the disease.^11-13^ The primary source of MMP-8 and -9 in the oral cavity is gingival crevicular fluid, an exudate secreted at the gingival margin.^14,15^ Mouthrinse sampling collects gingival crevicular fluid simultaneously from all the tooth-bearing sites in the mouth, proving a convenient method for assessing periodontal disease activity.^16,17^ A mouthrinse aMMP-8 point-of-care test has shown promise as a valuable tool for screening and evaluating treatment responses in periodontitis.^18-20^

Along with MMPs, cytokines, particularly interleukine-6 (IL-6), serve as pro-inflammatory mediators in periodontitis. Individuals with periodontitis exhibit elevated levels of IL-6 in their saliva and gingival crevicular fluid, reflecting its involvement in promoting immune responses and contributing to the resorption of periodontal tissue.^21^ RT induces inflammatory signaling and results in overexpression of interleukins, including IL-1β, IL-6, IL-8, TNF-α.^22,23^

Dysbiotic oral pathogens, such as *Porphyromonas (P.) gingivalis* and *Treponema (T.) denticola,* are commonly associated with chronic periodontitis.^24^ These bacteria produce proteases that are essential for the survival of these pathogens and that serve as virulence factors. The most important and well-studied classes of proteases of these pathogens are gingipains of *P. gingivalis* and dentilisin of *T. denticola*.^25,26^ These proteases can target host connective tissue proteins and facilitate the expression and activation of MMPs.^27^ The oral microbiome is influenced by RT and shifted to more periodontitis-associated flora.^28,29^ However, some studies have shown that RT of the head and neck area does not significantly affect the concentrations of *P. gingivalis* and *T. denticola* in saliva.^30,31^ To our knowledge, the impact of RT on the activities of bacterial proteases of *P. gingivalis* and *T. denticola* have not been studied to date.

This is a continuation of our previous research, which indicated deterioration of periodontal tissue and increased activation of MMP-8 and -9 in HNC patients receiving RT.^32,33^ The primary objective was to assess the impact of HNC RT in the biomarkers of periodontitis in mouthrinse samples and to reveal their possible associations with the progression of periodontitis. The second objective was to assess the impact on the proteases of oral periodontopathogens. Therefore, we assessed the biomarker levels before RT (T0) and at the end of RT (T1) and evaluated their associations with the change in CAL measurements between T0 and one month after RT had ended (T2) (Table 1). Based on the change in the average CAL across all teeth, we categorized the patients into two groups: those whose CAL was progressive (CAL ≥0.1 mm) and those who showed low/no progression (CAL <0.1 mm). Considering the deteriorating impact of HNC RT on oral tissues, we hypothesized that patients with HNC experience progression of periodontitis and changes in the biomarkers of periodontitis when receiving RT.

**Table 1.** Time-points for Mouthrinse Sampling and Periodontal Examination

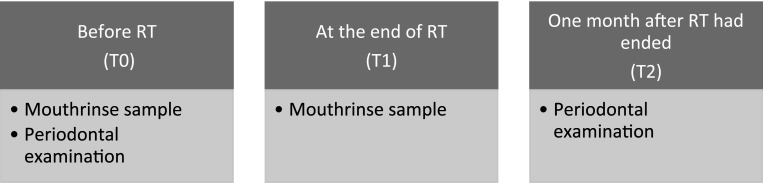

Abbreviations. RT = Radiotherapy; T = Time-point.

## Materials and Methods

### Patient Selection and Study Design

Twenty-four patients were recruited between years 2019-2021 from Başakşehir Çam and Sakura City Hospital, Istanbul, Turkey. Patients were selected randomly. The inclusion criteria were histologically confirmed HNC; at least 21 years of age; presence of at least five teeth; and RT with curative intent planned for HNC treatment. Patients with any previous cancer and/or RT, immune-associated disorders (i.e., chronic inflammatory diseases such as lupus erythematosus, rheumatoid arthritis, multiple sclerosis, Crohn’s disease, HIV); patients who received bisphosphonate therapy; and patients whose RT process was interrupted were excluded. Three patients had to be excluded due to missing mouthrinse samples. Therefore, twenty-one patients were finally included. All patient details were de-identified. Patients were guided to sustain good oral hygiene throughout their HNC treatment and to stop smoking.

The study was approved by the Biruni University Ethics Committee, Turkey (No: 2015-KAEK-67-22-05, date of approval 06.07.2022), and was conducted in accordance with the Declaration of Helsinki. All study participants signed an informed consent form. Demographic and medical data, and smoking habits were documented for each patient. Cancer therapy was planned individually by expert medical oncologists, in accordance with the National Comprehensive Cancer Network® guidelines without the influence of the current study. RT was administered for six weeks for each patient. The reporting of this study conforms to REMARK guidelines.^34^

### The Collection of Mouthrinse and Biomarker Analysis

Participants provided mouthrinse samples for periodontitis biomarker analysis at T0 and T1 (Table 1). Before sampling, the participants were instructed to refrain from eating or brushing their teeth for at least an hour before sampling. Participants first rinsed their mouths with tap water for 30 seconds, then spit it out. After 60 seconds, they rinsed with 5 mL of sterile purified water for 30 seconds and the sample was collected in a measuring cap.

For aMMP-8 analysis, mouthrinse samples were drawn from the measuring cap using a syringe, filtered, and transferred to the aMMP-8 point-of-care kit (Periosafe®). The quantitative analysis was conducted using the digital reader (ORALyzer®) following the manufacturer’s instructions. The minimum detectable concentration of aMMP-8 was set at 20 ng/ml in which the sensitivity and specificity is 76-90%.^19^ The remaining mouthrinse was moved into Eppendorf tubes and preserved at −70°C for laboratory analysis.

### Periodontal Clinical Examination

Periodontal examination was conducted twice for each patient by a one periodontist (MK). The first clinical examination and mouthrinse sampling was conducted 1-2 days before the first dose of RT at T0 and repeated one month after the RT had ended (T2) according to the hospital’s treatment protocol (Table 1). Periodontal clinical examinations were conducted for full-mouth using a 10-mm round-tip manual William’s periodontal probe. All permanent teeth, excluding the third molars, were examined at six sites for each tooth. Number of teeth present/missing, bleeding on probing, periodontal pocket depth, and CAL were recorded. Periodontal diagnosis was assessed with a new classification for periodontal diseases.^35^ The progression of periodontitis from T0 to T2 was assessed by measuring the change in the average CAL across all teeth. The patients were categorized into two groups: those whose CAL was progressive (CAL ≥0.1 mm) and those who showed low/no progression (CAL <0.1 mm).

### Laboratory Analysis

The concentrations of the total MMP-8 and -9, and IL-6 in the mouthrinse samples were detected using a commercially available enzyme-linked immunosorbent assay (ELISA) kit, following the manufacturer’s instructions (Human MMP-8/MMP-9/ IL-6 Quantikine® ELISA kit, R&D Systems, Minneapolis, MN, USA). The detection limits were 0.013 ng/ml for total MMP-8, 0.156 ng/ml for total MMP-9, and 0.70 pg/ml for IL-6. The presence of the gelatinases, particularly active MMP-9, in mouthrinse sample was assayed by gelatin-substrate zymography, using a previously described techique.^36^

### Substrate Design and Proteolytic Profiling

Fluorescence resonance energy transfer (FRET) peptide substrates specific for the detection of gingipain and dentilisin were designed based on cleavage preferences listed in the MEROPS peptidase database.^37^ Substrates were N-terminally flanked with a aminohexanoic acid (Ahx) linked fluorescein isothiocyanate (FITC) and C-terminally flanked with a lysine coupled quencher (KDabcyl). The substrates used in this study, gingipain (FITC-Ahx-RR-KDabcyl) and dentilisin (FITC-Ahx-AAPF-KDabcyl), were synthesized as described previously.^38^ Proteolytic activity was analyzed in mouthrinses of seventeen patients as described earlier.^38^ In brief, 49 μL sample was incubated with 1 μL 800 µM substrate and the increase in fluorescence was monitored using a fluorescence microplate reader (FLUOstar Galaxy, BMG Laboratories, Offenburg, Germany) with an excitation wavelength of 485 nm and an emission wavelength of 530 nm. Substrate cleavage was calculated by the fluorescence (F) emitted per minute for each sample. The slope was determined over the time intervals 0-20 minutes for gingipain and 0-10 minutes for dentilisin. A result was considered positive if the proteolytic activity reached or exceeded 5.0 F/min.

### Calculation of Cumulative Risk Scores (CRS)

The CRS analysis was first introduced as a novel periodontitis risk categorization model by Gursoy et al.^39^ The key in this approach is to utilize multiple biomarker data and diminish the number of false negatives. For the calculation of CRS, the mouthrinse concentrations of MMP-8, -9, and IL-6, which were separately analyzed in our previous studies,^32,33^ were statistically re-analyzed together with new data of bacterial proteolytic activity. Each biomarker was assigned a value of 1 or 2 based on whether its mouthrinse concentration was below (1) or above (2) the median level for that biomarker. These sub-scores for a/t MMP-8 and -9, IL-6, dentilisin, and gingipain were then calculated by multiplying together in different combinations, resulting in combined CRS values at two time points, just before radiotherapy and at the end of the RT.

### Statistics

The data were statistically analyzed using SPSS version 29.0 (IBM SPSS Statistics for Windows, IBM Corp., Armonk, NY, USA). A *P*-value below 0.05 was considered statistically significant. We used RP ANOVA test to analyze the change in biomarker concentration between the two time points, as well as the association with CAL progression, categorized as either progressive (CAL ≥0.1 mm) or low/no progression (CAL <0.1 mm). aMMP-8 concentrations of ≥10 ng/ml were used accordingly in the calculations. For technical reasons, aMMP-8 values <10 ng/ml were marked as 5 in SPSS, representing an average between 0 and 10 ng/ml.

Previously, Keskin et al. have found a large effect size (larger than 0.40) related to aMMP-8 and periodontal degeneration due to HNC radiotherapy (eta-squared = 0.276 corresponding effect size f = 0.617).^5^ The sample size calculation was based on whether the change in concentration of the biomarker between T0 and T1 is related to the development of CAL destruction between T0 and T2 measurements. A sample size calculation for a repeated measures ANOVA (within-between interaction) was performed (G*Power 3.1), which revealed that a total of 16 patients were required to reach 80% power with a large effect size 0.40, a significance level of 5% and two groups and two repeated measurements, when using correlation among repeated measure of 0.5 and nonsphericity correction epsilon of 1.

## Results

Patient demographic, oncological, and periodontal characteristics are presented in Table 2, and in more detail in Brandt et al. 2024.^33^ Briefly, mean age was 55 years (range, 28-84), all patients were past or present heavy smokers (>10 cigarettes per day) and suffered from periodontitis. Most of the patients (71%) had moderate or severe (stage 2 or 3) periodontitis at T0. In addition, all patients had a risk for rapid periodontitis progression (grade C). The three most common HNC primary locations were nasopharynx (29%), larynx (19%), and oropharynx (14%). The HNC treatments varied among the patient group, and RT combined with either surgery (38%) or chemotherapy (38%) was the most common modality. A total RT dose received for each patient was on average 65 Gy, ranging from 50-70 Gy. Eleven (52%) patients experienced CAL progression (≥ 0.1 mm) while 10 patients (48%) showed low or no progression (< 0.1 mm) between T0 and T2.

**Table 2.** Patient Characteristics. The Top Section of the Table Describes the Patients Who Developed CAL-Progression of ≥0.1 mm on Average, While the Lower Section Lists the Patients Who Exhibited Low or No Progression in Their Mean CAL

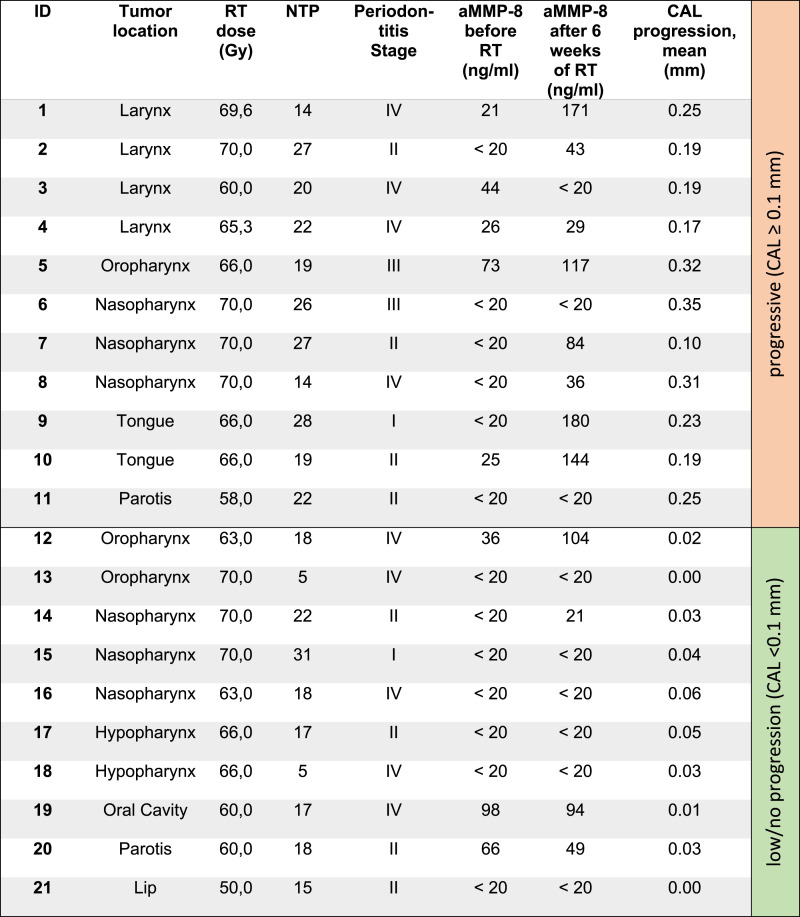

Abbreviations: RT = Radiotherapy; NTP = Number teeth present; aMMP = Active matrix metalloprotease; CAL = Clinical attachment loss.

**Figure 1.**
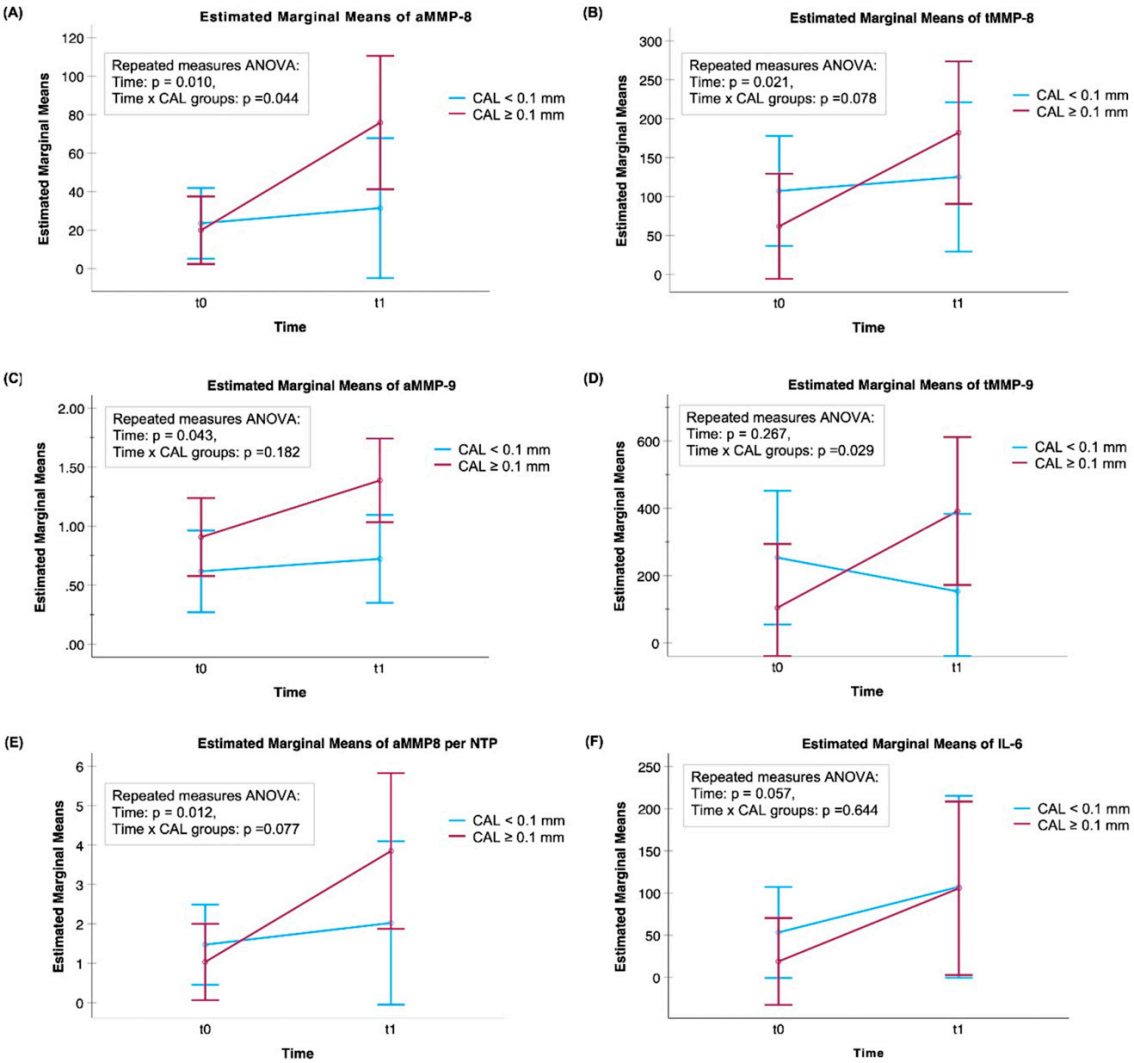
Mean Levels of (A) Active (a)MMP-8 (ng/mL), (B) Total (t)MMP-8 (ng/mL), (C) aMMP-9 (ng/mL), (D) tMMP-9 (ng/mL), (E) aMMP-8 (ng/mL) per the Number of Teeth Present (NTP), and (F) IL-6 (pg/mL) (n = 21) With 95% Confidence Interval Bars for the Time-points of t0 = before Radiotherapy (RT) and t1= at the End of RT. A Mixed ANOVA Analysis was Used in Calculating p-values. CAL ≥0.1 mm: progressive Periodontitis; CAL <0.1 mm: low/No Periodontitis Progression. Time = the Effect of RT in the Biomarker Concentrations. Time x CAL = The Association Between Changes in Biomarker Concentrations and the Development of CAL (CAL ≥0.1 mm or CAL <0.1 mm)

**Figure 2.**
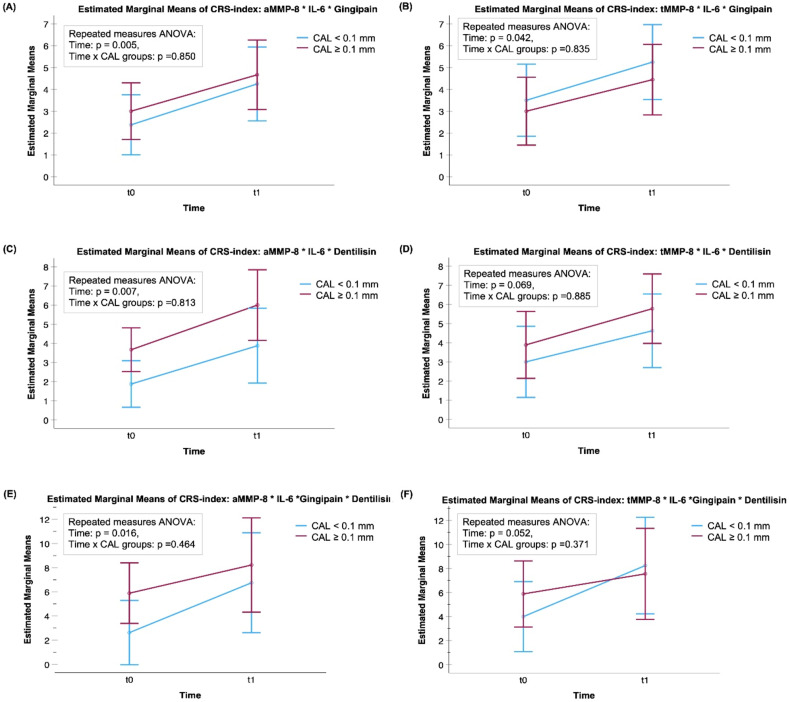
Mean Levels of Modified CRS Indices Based on (A) Active (a)MMP-8 * IL-6 * Gingipain Activity, (B) Total (t)MMP-8 * IL-6 * Gingipain Activity, (C) aMMP-8 * IL-6 * Dentilisin Activity, (D) tMMP-8 * IL-6 * Dentilisin Activity, (E) aMMP-8 * IL-6 * Gingipain Activity * Dentilisin Activity, and (F) tMMP-8 * IL-6 * Gingipain Activity * Dentilisin Activity With 95% Confidence Interval Bars for the Time-points of t0 = before Radiotherapy (RT) and t1= at the End of RT. A Mixed ANOVA Analysis was Used in Calculating p-values, n = 17. CAL ≥0.1 mm: progressive Periodontitis; CAL <0.1 mm: low/No Periodontitis Progression. Time = the Effect of RT in the Biomarker Concentrations. Time x CAL = The Association Between Changes in Biomarker Concentrations and the Development of CAL (CAL ≥0.1 mm or CAL <0.1 mm). * Indicates Multiplying

**Figure 3.**
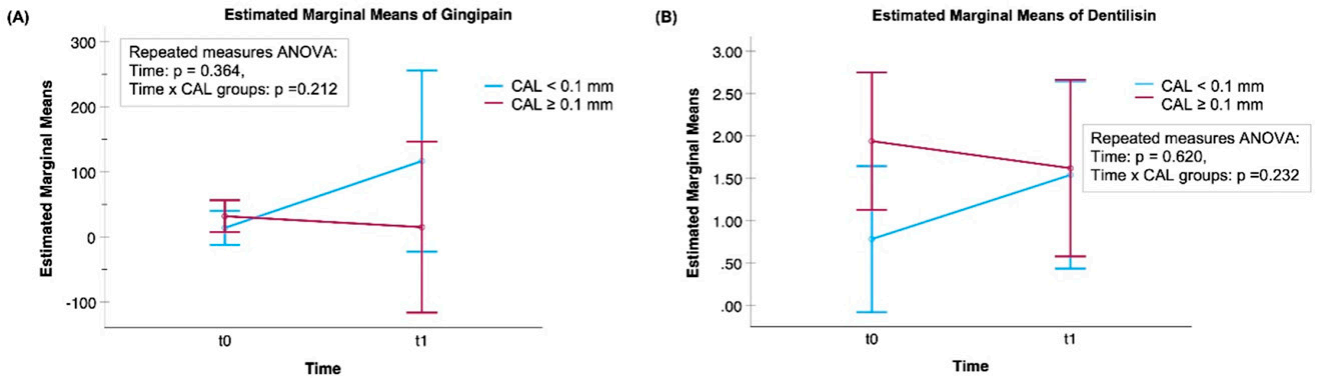
Mean Levels of (A) Gingipain Activity and (B) Dentilisin Activity (n = 17) With 95% Confidence Interval Bars for the Time-points of t0 = before Radiotherapy (RT) and t1 = at the End of RT. A Mixed ANOVA Analysis was Used in Calculating p-values. CAL ≥0.1 mm: progressive Periodontitis; CAL <0.1 mm: low/No Periodontitis Progression. Time = the Effect of RT in the Biomarker Concentrations. Time x CAL = The Association Between Changes in Biomarker Concentrations and the Development of CAL (CAL ≥0.1 mm or CAL <0.1 mm)

The mean levels of aMMP-8, aMMP-8 per the number of teeth present (NTP), tMMP-8, aMMP-9, and four modified CRS-indices significantly increased during RT (*P* < 0.05) (Figures 1 and 2). aMMP-8 and tMMP-9 were the only biomarkers with mean levels associated with CAL progression (*P* = 0.044, *P* = 0.029, respectively) (Figure 1). For those who had CAL progression of 0.1 mm or more, i.e. clinical progression of periodontitis, the mean levels of aMMP-8 and tMMP-9 increased significantly during RT (*P* = 0.019 and *P* = 0.026, respectively). In the group of patients with low/no progression of CAL (< 0.1 mm), there were no significant changes in aMMP-8 or tMMP-9 levels (*P* = 0.303, *P* = 0.431, respectively). Concerning bacterial proteases, there were no significant effects of the RT in the mean levels of activities of gingipain (*P* = 0.365) or dentilisin (*P* = 0.620) nor were there any associations with the progression of CAL (Figure 3). None of the modified CRS indices showed any significant associations with the progression of CAL in their mean levels (Figures 1–3).

## Discussion

In this study, we present a novel biomarker-based approach to assess the progression of periodontitis in patients receiving RT for HNC. Traditionally, the assessment of periodontal health involves the use of a periodontal probe, which is invasive, sometimes painful for patients, and may lead to bacteremia.^40^ Therefore, it would be beneficial to assess periodontal health by using biomarkers in oral fluids. Mouthrinse contains valuable markers for periodontal inflammation and is easy and non-invasive to collect, making it suitable for biomarker approaches.^15^ Importantly, the application of mouthrinse sampling and biomarker analysis could be conveniently integrated into clinical protocols for the dental examination of patients with HNC. Mouthrinse sampling and aMMP-8 testing can be easily performed by any healthcare staff or even by the patient. During a pre-treatment dental evaluation, utilizing aMMP-8 point-of-care testing could provide additional insights into the current activity of periodontitis, thereby complementing the clinical periodontal assessment. After RT, aMMP-8 testing could screen for the progression of periodontitis without requiring a dental appointment. The goal of screening biomarkers is to identify patients who are at high risk for the progression of periodontitis, ideally before the clinical progression appears. This would benefit the patients with early diagnostics of periodontitis and offer the opportunity to implement strategies that minimize clinical manifestations.

In this study, we assessed the periodontitis progression by observing the change of average CAL across all teeth one month after RT, compared to the measurements prior to RT. In the recently updated periodontitis classification protocol, CAL is the key factor determining the severity and clinical disease activity of periodontitis, with CAL ≥2 mm in 5 years considered rapid progression of the disease.^35^ In the present study, the CAL progression limit was established at 0.1 mm, categorizing progressive development with CAL ≥0.1 mm and low/no progression with CAL <0.1 mm. This threshold was set not necessarily due to its clinical significance, but rather to demonstrate the capacity of each biomarker to detect even minimal clinical progression in periodontitis and test sensitivity of the biomarkers. A change in CAL at a specific site of less than 0.1 mm cannot be detected through clinical observation. Nevertheless, this calculation was based on the average change in CAL across all teeth, thereby enhancing the reliability of the results and offering an opportunity to identify early clinical signs of the progression of periodontitis. Interestingly, this study reveals that aMMP-8 and tMMP-9 can identify the progression of active periodontitis even at such early stages.

This study is the first to demonstrate that the changes in the mean levels of aMMP-8 and tMMP-9 seem to associate with the progression of CAL among patients with HNC receiving RT. Notably, a positive aMMP-8 point-of-care test result at T1, with the cut-off of 20 ng/ml, was able to predict CAL progression at T2. Thus, the aMMP-8 point-of-care test could be used at the end of HNC RT to identify patients at higher risk for the progression of periodontitis. Indeed, our findings suggest that collagenolytic aMMP-8, not tMMP-8, is linked to the progression of CAL. This aligns with earlier studies indicating that aMMP-8 serves as a more reliable marker for identifying progressive periodontitis compared to tMMP-8.^41-43^ Similarly, regarding MMP-9, the association of tMMP-9 with CAL progression further strengthens its ability to predict periodontal disease activity.^44^

Although previous studies have shown that RT of the head and neck area causes the elevation of IL-levels in oral fluids,^5,23,45^ the present study demonstrated no significant impact on IL-6 levels due to RT (*P* = 0.057). In addition, despite four of the six modified CRS indices showing significant effects due to HNC RT, they were not associated with CAL progression. Therefore, in our study, this combined biomarker analysis method did not enhance the clinical relevance of detecting progressive periodontitis compared to a single biomarker.

In the present study, we show quantitatively, for the first time to our knowledge, that the activities of *T. denticola’s* dentisilin and *P. gingivalis*’ gingipain are not significantly affected by RT. However, it is possible that their concentrations in mouthrinse are too low to determine the differences during RT. Gingival crevicular sampling at individual sites using sterile paper points and/or micropipettes could provide more concentrated sample in this regard.^46^

While RT has an irreplaceable therapeutic role in the management of HNC, it results in inflammation, changes in immunity, and alterations in the oral microbiome within the irradiated tissues.^2,28^ In addition, cytotoxic effects of RT lead to compromised wound-healing capacity and impaired tissue regeneration, which may result decreased integrity of periodontal tissues.^28^ Periodontitis results from a complex interaction between microbial pathogens and the host’s immune response, both of which can be influenced by RT. Our results showing the progression of periodontitis in patients with HNC receiving RT are in line with previous clinical studies.^4,5,47^

Several factors triggered by RT may lead to increased levels of MMP-8 and -9. Indeed, proinflammatory pathways and heightened cytokine levels along with a shift toward a more dysbiotic microbiome have all been implicated in the increased expression and activity of MMP-8 and -9.^8,48,49^ Although tissues undergo oxidative stress during RT, the role of most reactive oxygen species (ROS) as in vivo activators of MMPs has not been clearly established.^50^ Additionally, the activity of MMPs is closely linked to the balance between the levels of MMPs and the tissue inhibitors of metalloproteinases (TIMPs).^11^ However, our earlier pilot study findings showed that HNC RT does not have a significant effect on the TIMP-1, which is the most abundant inhibitor of both MMP-8 and -9.^33^

All patients were past or present heavy smokers and had periodontitis when diagnosed with HNC. Therefore, we propose that RT induces and triggers the existing periodontitis to proceed to the rapid progression. In this rather small and heterogeneous patient group, we were unable to distinguish why approximately half of the study population experienced rapid CAL progression while the others showed low or none. However, we anticipate that periodontitis progression occurs later in all patients who present with existing periodontitis, significant history of smoking, and RT side effects affecting the oral cavity. In addition, future research with extended time intervals could provide valuable insights into whether early rapid progression of periodontitis and heightened levels of aMMP-8 or tMMP-9 are indicative of continued rapid progression over a longer duration.

We have previously demonstrated that the levels of MMP-8 and MMP-9 reach their peak at the conclusion of RT and then begin to decline.^33^ Therefore, we utilized T0 and T1 to capture the maximum impact of RT on these biomarkers. In the present study, clinical periodontal data were collected at T0 and T2 as these dental evaluations are routinely included in the hospital’s treatment protocol. Conducting a periodontal examination at T1 would not benefit the patient and would, in fact, be an unnecessarily invasive procedure. While obtaining periodontal data at T1 would have offered additional insights into the relationship between simultaneous biomarker levels and clinical status, the periodontal assessment performed at T2 is still relatively close to the peak of biomarker levels at T1.

We acknowledge that this pilot study has certain limitations. Although we reported significant findings, it’s important to emphasize that this is an observational pilot study, and the results should be regarded as preliminary. There were variations in the primary sites of carcinomas, treatments, RT doses, and primary targets. The impact of patients’ medical history on CAL progression should be assessed in a larger study group. Having a control group of HNC patients who do not undergo RT would provide valuable insights into how alternative treatments affect biomarker levels and clinical periodontal status. Surgery, whether performed alone or in combination with tissue reconstruction or concomitant chemotherapy, may independently affect the concentrations of oral fluid biomarkers. Data on the severity of oral mucositis could provide valuable insights into how concurrent inflammatory conditions affect biomarker levels detected in mouthrinse. Nevertheless, the key strength of this study is periodontal examinations conducted by a single experienced periodontologist. This way we can ensure that clinical examinations at T0 and T2 are reliably comparable with each other, as inconsistencies would weaken the reliability of the results. In addition, the strengths include having an appropriate sample size according to power calculations. Furthermore, the treatment plans for carcinoma align with current clinical guidelines.

## Conclusions

RT in head and neck area appears to induce the rapid progression of periodontitis. This progression may be driven by increased activations of MMP-8 and -9 due to HNC RT. Furthermore, HNC RT may not significantly impact the activities of the bacterial proteases dentilisin or gingipain. A clinically available, non-invasive aMMP-8 point-of-care test could be a valuable clinical tool to detect the progression of periodontitis at the end of HNC RT and thus signal the need for preventive dental hygiene actions. Further research into the progression of periodontitis induced by HNC RT, early diagnostics with biomarkers, and potential prevention methods are important areas for future studies.

## Data Availability

Due to the sample size of the study and to conserve participant confidentiality and anonymity, the authors elect not to share data. *

## Appendix

### Abbreviations

MMP: Matrix metalloproteinases
HNC: Head and neck carcinoma
RT: Radiotherapy
ELISA: Enzyme-linked immunosorbent assay
aMMP: active MMP
tMMP: total MMP
T: Time-point
FRET: Fluorescence Resonance Energy Transfer
CRS: Cumulative risk score
CAL: Clinical attachment loss
IL: Interleukine
P. gingivalis: Porphyromonas gingivalis
T. denticola: Treponema denticola
FITC: Fluorescein isothiocyanate
F: Fluorescence
NTP: Number teeth present
